# Non-Invasive Diagnostic Tests for Portal Hypertension in Patients with HBV- and HCV-Related Cirrhosis: A Comprehensive Review

**DOI:** 10.3390/medicina60050690

**Published:** 2024-04-24

**Authors:** Ciro Celsa, Marzia Veneziano, Francesca Maria Di Giorgio, Simona Cannova, Antonino Lombardo, Emanuele Errigo, Giuseppe Landro, Fabio Simone, Emanuele Sinagra, Vincenza Calvaruso

**Affiliations:** 1 Gastroenterology and Hepatology Unit, Department of Health Promotion, Mother & Child Care, Internal Medicine & Medical Specialties, University of Palermo, 90127 Palermo, Italy; veneziano.marzia@gmail.com (M.V.); francescam.digiorgio@libero.it (F.M.D.G.); simicannova@hotmail.it (S.C.); lombanino@gmail.com (A.L.); emanuele.errigo97@gmail.com (E.E.); peppe.landro@virgilio.it (G.L.); simone.f65@libero.it (F.S.); vincenza.calvaruso@unipa.it (V.C.); 2 Department of Surgery & Cancer, Imperial College London, Hammersmith Hospital, London W12 0NN, UK; 3 Gastroenterology and Endoscopy Unit, Fondazione Istituto G. Giglio, 90015 Cefalù, Italy; emanuelesinagra83@googlemail.com

**Keywords:** portal hypertension, non-invasive tests, liver stiffness, spleen stiffness, hepatic decompensation, cirrhosis

## Abstract

Clinically significant portal hypertension (CSPH) in patients with compensated advanced chronic liver disease indicates an increased risk of decompensation and death. While invasive methods like hepatic venous–portal gradient measurement is considered the gold standard, non-invasive tests (NITs) have emerged as valuable tools for diagnosing and monitoring CSPH. This review comprehensively explores non-invasive diagnostic modalities for portal hypertension, focusing on NITs in the setting of hepatitis B and hepatitis C virus-related cirrhosis. Biochemical-based NITs can be represented by single serum biomarkers (e.g., platelet count) or by composite scores that combine different serum biomarkers with each other or with demographic characteristics (e.g., FIB-4). On the other hand, liver stiffness measurement and spleen stiffness measurement can be assessed using a variety of elastography techniques, and they can be used alone, in combination with, or as a second step after biochemical-based NITs. The incorporation of liver and spleen stiffness measurements, alone or combined with platelet count, into established and validated criteria, such as Baveno VI or Baveno VII criteria, provides useful tools for the prediction of CSPH and for ruling out high-risk varices, potentially avoiding invasive tests like upper endoscopy. Moreover, they have also been shown to be able to predict liver-related events (e.g., the occurrence of hepatic decompensation). When transient elastography is not available or not feasible, biochemical-based NITs (e.g., RESIST criteria, that are based on the combination of platelet count and albumin levels) are valid alternatives for predicting high-risk varices both in patients with untreated viral aetiology and after sustained virological response. Ongoing research should explore novel biomarkers and novel elastography techniques, but current evidence supports the utility of routine blood tests, LSM, and SSM as effective surrogates in diagnosing and staging portal hypertension and predicting patient outcomes.

## 1. Introduction

Portal hypertension (PH) is a major complication of cirrhosis and it represents one of the main mechanisms leading to clinical events of decompensation (ascites, oesophageal varices [OV], and hepatic encephalopathy) that are associated with considerable morbidity and mortality. Portal hypertension is usually diagnosed according to the gradient between the portal vein and the inferior vena cava—the hepatic venous pressure gradient (HVPG), that normally ranges from 1 to 4 mmHg, with values greater than 5 mm Hg indicating portal hypertension and values equal to or greater than 10 mm Hg corresponding to clinically significant portal hypertension (CSPH). Particularly, HVPG is the gradient between the sinusoidal pressure (wedged hepatic venous pressure) and the hepatic venous pressure (free hepatic venous pressure). The wedged hepatic vein pressure is measured via balloon occlusion of the hepatic vein, while free hepatic venous pressure is measured in the hepatic vein within 2–3 cm of its confluence with the inferior vena cava [1]. In patients with cirrhosis, anatomical changes in the intrahepatic circulation produce growing portal venous resistance, while splanchnic vasodilatation and the increased cardiac output raise the portal venous flow, resulting in PH. HVPG is the gold standard in detecting CSPH, but it is necessary to consider that the presence and the severity of intrahepatic shunts could underestimate the wedged hepatic vein pressure; ideally, the portal vein should be catheterized to measure portal pressure in these cases [2]. Moreover, invasiveness of this technique (the insertion of a balloon-tipped catheter into the right internal jugular vein to reach right atrium, inferior vena cava, and the hepatic vein; the flow occlusion; the use a small volume of contrast medium; and the sedation of the patient, which is required in some situations) is a reason for limited use in clinical practice.

The natural history of hepatitis B virus (HBV) and hepatitis C virus (HCV) infection includes necroinflammation, fibrosis, and alterations in liver microcirculation, which represent a key mechanism for the development of PH and its complications.

**Figure 1:**
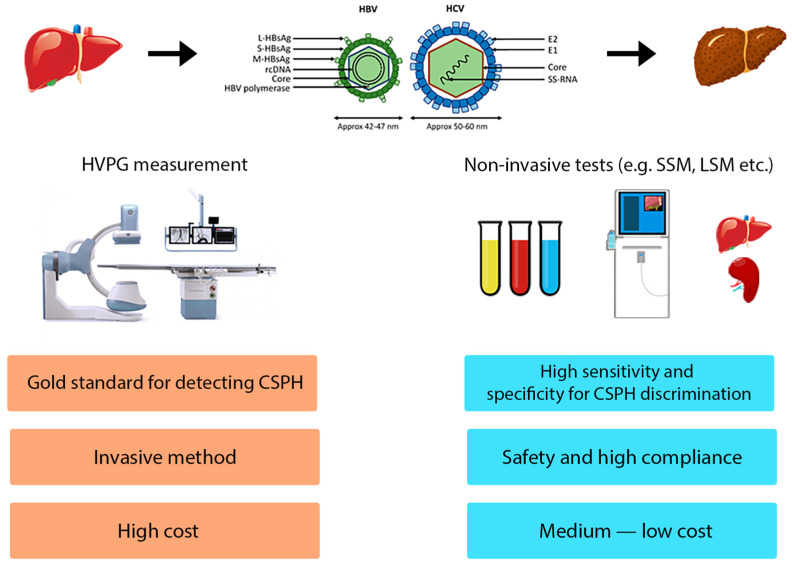
Tools for evaluation of CSPH.

Non-invasive tests (NITs) can help in the diagnosis of CSPH and in the follow-up of patients with HBV- or HCV-related compensated advanced chronic liver disease (cACLD) and CSPH, that represents a population at high risk of hepatic decompensation (Figure 1).

Biochemical-based NITs can be represented by single serum biomarkers or by composite scores, these latter derived from prediction models. Single serum markers include platelet (PLT) count, aspartate transaminase (AST), alanine aminotransferase (ALT), albumin, gamma-glutamyl transferase (GGT), bilirubin, or international normalized ratio (INR). Single serum markers can be combined with each other or with demographic characteristics to obtain composite scores, such as the Fibrosis-4 (FIB-4) Index for Liver Fibrosis [3,4] or AST/platelet ratio index (APRI) [5].

On the other hand, liver stiffness measurement (LSM) and spleen stiffness measurement (SSM) can be assessed using a variety of instrumental techniques, and they can be used alone, in combination with, or as a second step after biochemical-based NITs. NITs are able to predict CSPH and could correlate with HVPG, resulting in a better follow-up of patients and in an optimization of treatment via disease-modifying agents [6] (e.g., non-selective beta-blockers).

### NITs Evaluation of Diagnostic Accuracy

When evaluating a non-invasive prediction rule, several considerations should be taken into account. First of all, NITs should be assessed in terms of diagnostic performance. Most published studies have usually evaluated NITs in terms of conventional operating characteristics, such as sensitivity, specificity, and false positive and negative rate. These measures are usually summarized by AUROC (area under the receiver operating characteristic), which is a measure of discrimination, i.e., the ability of a test of correctly discriminate between the target condition and its absence (for example, the presence or the absence of CSPH or high-risk varices (HRV)). More rarely, longitudinal studies also report information about the calibration of a test, which is the evaluation of the difference between the observed and predicted outcomes. In other words, calibration measures the degree of how the application of a prediction rule could over- or underestimate the probability of an event. Physicians and researchers should always focus not only on the discrimination but also on the calibration, because two models could have the same AUROC (hence, the same discriminating ability) but different calibration (hence, one could overestimate the risk of the event of interest, while the other could underestimate). However, it should be considered that both traditional statistical metrics, such as discrimination and calibration, do not incorporate the clinical consequences of a decision based on a model or a test. Decision curve analysis can overcome these limitations, given that this methodology is able to calculate the net clinical benefit associated with the use (or not) of a test, weighting the risk of a false negative with that of a false positive and allowing for the interpretation of the different clinical consequences that a false negative (or a false positive) test results could have in different clinical settings [7]. More importantly, decision curve analysis can also measure the net benefit of a test at different risk thresholds of missing the event of interest, so it could be an ideal methodology to assess and compare NITs in the setting of the prediction of PH. Particularly, it has been demonstrated that NITs including LSM (i.e., Baveno VI criteria) or solely based on biochemical tests (i.e., Rete Sicilia Selezione Terapia-HCV (RESIST-HCV) criteria, including PLT and albumin) are associated with a significant net benefit when used in patients with HCV-related cACLD, for the prediction of HRV both at the time of starting direct-acting antivirals (DAAs) and after sustained virological response (SVR) [8,9].

In addition to methodological considerations, the local availability of tools included in NITs should also be taken into account. We discuss the utility of LSM and SSM using different techniques for the risk stratification in the setting of cACLD. However, these tools are not universally available, especially in low-resource settings or outside tertiary referral centers. While new research efforts have to be made to identify novel tools to non-invasively assess PH based on innovative technologies or methodologies (e.g., machine or deep learning), a simplification consisting of the use of easily and universally available NITs with well-demonstrated clinical utility and benefit is needed to optimize the management of patients with cACLD in clinical practice and in different healthcare settings.

## 2. Overview of Non-Invasive Tests for Predicting PH

### 2.1. Liver Stiffness Measurement (LSM)

#### 2.1.1. Transient Elastography (TE)

LSM via vibration-controlled transient elastography (VCTE) is the most commonly employed tool in clinical practice to stage the severity of fibrosis and it has a well-recognized role in the risk stratification of PH [10]. This tool is useful to quantitatively assess liver fibrosis and steatosis in patients with liver disease: it measures the speed of shear waves generated by a push pulse and its propagation that varies depending on liver texture (faster in hard liver tissue than in soft liver tissue). TE uses the median value of at least 10 valid measurements as the representative stiffness value, and several factors can affect liver stiffness, including nonfasting status, high transaminases levels, cardiac congestion, or cholestasis. A prospective study on 13,369 TE examinations showed that body mass index (BMI), lower operator experience, older age, female gender, and metabolic factors were associated with a higher risk of unreliable results [11]. Two different probes (M and XL) can be used according to anthropometric characteristics of patients: M is used as the first step, while the XL probe can be used in case of no valid shot or unreliable measurement, which are common in obese patients [12].

LSM is important to discriminate between patients with and without CSPH: in a meta-analysis of 11 studies, the hierarchical summary AUROC for CSPH discrimination was 0.90 (sensitivity 87.5%, 95% CI 75.8–93.9%; specificity 85.3%, 95% CI 76.9–90.9%) and the summary HVPG-LSM correlation coefficient was 0.783 (95% CI 0.737–0.823) [13].

In combination with PLT count, LSM via TE is recommended by Baveno VI consensus to identify patients with a low risk of having HRV, i.e., those with LSM < 20 kPa and PLT count > 150,000/mmc [14]. The application of these criteria allows us to avoid 15–40% of unnecessary oesophagogastroduodenoscopy (OGD) procedures with 26–46% specificity.

Moreover, Baveno VII consensus suggests the use of persistent values of LSM < 12 kPa and PLT count > 150,000/mmc in patients with cirrhosis who achieve SVR to exclude CSPH and to identify a low risk of decompensation [15]. Furthermore, LSM and PLT count can rule out HRV in patients with HCV- and HBV-related cirrhosis who achieved SVR and viral suppression, resulting in being discharged from portal hypertension surveillance.

Recent guidelines support the non-invasive diagnosis of CSPH in patients with VCTE > 20–25 kPa in virus-related cirrhosis and suggest that VCTE > 20–25 kPa should be used for CSPH risk stratification in cACLD, ideally in combination with PLT count and spleen size [16]. According to the results of the ANTICIPATE study that involved 518 patients with cACLD with paired NITs, Baveno VII consensus proposed rule-out criteria for CSPH, represented by LSM ≤ 15 kPa and PLT count ≥ 150,000/mmc [17].

Nevertheless, it is important to highlight that co-existence of metabolic factors such as obesity could reduce the accuracy of LSM, requiring that its values should be interpreted together with clinical and biochemical data and with controlled attenuation parameter (CAP) values, a surrogate of the severity of steatosis [18].

#### 2.1.2. Shear Wave Elastography (SWE)

LSM via VCTE remains a point-of-care method, but techniques like Virtual Touch Quantification (VTQ) via acoustic radiation force impulse (ARFI) imaging, point shear wave elastography (pSWEs), and supersonic shear wave elastography are able to assess, in real time, the region of interest where elasticity has to be measured and to obtain a semi-quantitative assessment of elasticity via color-coding and quantitative measurement (m/s or kPa). Table 1 summarizes the differences between the main elastography-based techniques.

Differently from VCTE, which employs mechanical impulse, SWE uses an acoustic radiation force impulse to induce shear waves in liver tissue. It can be performed simultaneously with ultrasonography, because it is included in a conventional ultrasonographic system with a 4.5 MHz curved probe. This technique is based on the shearing of the examined tissue that induces a smaller strain in hard tissues than in soft ones. The probe automatically produces an acoustic ‘push’ pulse that results in the generation of shear-waves, for which propagation speed is measured in m/s and then converted into kPa to estimate the stiffness. It seems to be more accurate than TE because it measures the displacement by comparing the locations of tissue echoes emitted before and after the impulse and it is not limited by obesity, ascites, and narrow intercostal spaces, resulting in a significantly lower probability of unreliable measurements compared to TE (2.1% vs. 6.6%, respectively) [19].

#### 2.1.3. Magnetic Resonance Elastography (MRE)

Magnetic resonance elastography (MRE) is a tissue stiffness assessment technique that is used to evaluate liver fibrosis as a less invasive alternative to liver biopsy. Compared to other techniques such as TE, it has the advantage of not depending on the operator carrying out the exam. It is certainly a longer and more expensive procedure than the alternatives; therefore, its use in clinical practice remains limited. The mechanical waves are sent to the passive driver through a connecting tube that is placed on the external abdominal wall, across the chest or the right lobe of the liver. The exam is based on the “MRE sequence”: the propagating shear waves are integrated with modified phase-contrast, gradient-echo sequence and cyclic motion-encoding gradients sensitive to though-plane motion. By processing magnitude and phase images and using an algorithm, it is possible to calculate the tissue visco-elasticity (kPa). The liver is divided into free-hand region of interest and it is possible to avoid regions of wave interference (e.g., large vessels, dilatated bile ducts, and artifacts; moreover, it is not affected by ascites, obesity, and interposition of bowel as for biopsy and LSM). The cut-off values for detecting liver fibrosis any, significant, cirrhosis, and chronic liver disease are 3.45, 3.66, 4.11, and 4.71 kPa; this tool is accurate in detecting early stages of fibrosis: the sensitivity and specificity in assessing all grades of liver fibrosis is greater than 95%, because MRE could detect heterogeneous patterns of fibrosis and early fibrotic changes, that could be missed by biopsy or LSM due to the limited sampling area. On the other hand, MRE requires patient’s respiratory cooperation and it could not be performed in patients with claustrophobia. As for technical limitations, iron overload results in MRI signals that are too low and the process of postproduction of elastography is longer than that of LSM [20]. A small prospective study by Gharib et al. included 23 subjects with different aetiologies (nine patients with HCV mono-infection, nine with HCV/Human Immunodeficiency Virus (HIV) co-infection, and five with HIV/Non-Alcoholic Steatohepatitis (NASH)), showing that MRE was able to predict HVPG [21]. To date, there is not much data available that correlates MRE with CSPH, especially in viral aetiologies. It would be very interesting to better understand the clinical utility of MRE to optimize the follow-up of patients at high risk of developing CSPH.

### 2.2. Spleen Stiffness Measurement (SSM)

#### 2.2.1. Transient Elastography

PH determines spleen parenchymal remodeling, due to passive congestion increased arterial inflow, and increased splenic lymphoid tissue, with higher angiogenesis and fibrogenesis, resulting in splenomegaly and changes in stiffness: the spleen is stiffer in patients with liver disease than in healthy patients [22]. SSM is related to PH and extra-hepatic hemodynamic changes and passive congestion hyperplasia, angiogenesis, and fibrogenesis of the spleen are associated with increased SSM values. Differently from LSM, which is able to assess liver fibrosis and increased intrahepatic vascular resistance and is a good surrogate for liver biopsy, SSM can predict CSPH because of its correlation with PH and splanchnic haemodynamic changes [22].

Early studies investigating SSM have employed the standard VCTE device and probe used for LSM, with a fixed shear wave frequency at 50 Hz (SSM @50 Hz), adapted for a depth and a stiffness range between 1.5 and 75 kPa. However, the location and the physical characteristics of the spleen can result in an overestimation of spleen stiffness using this device and its upper limits affect the possibility of a precise assessment of SSM. For these reasons, a new spleen-dedicated VCTE setting for the M probe was introduced, with a fixed shear wave frequency at 100 Hz (SSM @100 Hz) [23].

In patients with HCV-related cirrhosis, SSM has been demonstrated to be able to accurately identify patients with OV and CSPH [24] and to be correlated with OV size, when using SSM@100 Hz [25]. Moreover, SSM is also an accurate predictor of clinical decompensation of cirrhosis [26].

For patients who do not meet the Baveno VI criteria, Baveno VII consensus recommended to use the SSM value < 40 kPa to avoid more OGD with a missed HRV rate < 5%.

A single-center, cross-sectional study conducted in Japan compared LSM, SSM@50 Hz, and SSM@100 Hz with HVPG in predicting OV. Vibration-controlled transient elastography was developed for the liver with a fixed shear wave frequency at 50 Hz (SSM@50 Hz), adapted for a depth and a stiffness range between 1.5 and 75 kPa, then a new spleen-dedicated VCTE setting for the M probe was created, with a fixed shear wave frequency at 100 Hz (SSM @100 Hz). The authors showed SSM was more reliable than LSM for the diagnosis of OV with an AUROC of 0.933 for SSM@100 Hz; moreover, SSM@50 Hz and SSM@100 Hz showed a significant correlation with HVPG and SSM@100 Hz had an higher specificity (82%) in detecting HRV than SSM@50 Hz (67.1%). In this study, LSM had a lower correlation with PH than SSM because of the inability to recognize the hemodynamic change characteristic of hyperdynamic syndrome and the presence of portosystemic shunts [27]. A multicenter European prospective study with 260 cirrhotic patients showed a correlation between HVPG and SSM@100 Hz and a better diagnostic accuracy of SSM@100 Hz in predicting OV, large OV, and HRV compared to other NITs like LSM and SSM@50 Hz. SSM@100 Hz with a cut-off ≤ 41.3 kPa in addition to the Baveno VI recommendation spared 38.9% OGD with a missed HRV rate < 5% [23].

More recently, an individual patient data meta-analysis including 1245 patients from 17 studies showed that the combination of SSM with the Baveno VII algorithm is effective in reducing the proportion of patients falling in the grey zone classification for CSPH, especially when using a single cut-off model [28].

Comparing LSM and SSM, the data show that spleen stiffness (SS) might be a superior marker of PH compared with LSM in patients with cirrhosis of viral aetiology, and a growing body of evidence suggests that this might also be true for other aetiologies. A recent study realized by Ravaioli et al. also showed that, compared with LSM, measurement of SSM is a direct surrogate for PH in patients after recovery from HCV and could be useful for monitoring responses and stratifying risk after therapy, suggesting that SSM might be a more dynamic marker, reflecting “acute” changes in HVPG not detected via LS [29].

#### 2.2.2. Shear Wave Elastography (SWE)

A cross-sectional, prospective, single-center study of about 60 patients with liver cirrhosis, analyzed the correlation between LSM and SSM (assessed using ARFI) and HVPG [30]. The patients underwent ultrasound (US) examination (with LSM and SSM assessed using ARFI), HVPG, OGD, and blood examination and the authors found a strongly linear association between SSM, LSM, and HVPG. The coefficient between SSM and HVPG was better than the one between LSM and HVPG (*p* < 0.0001) in patients with HVPG of 10 mmHg and over, while there was a significant correlation between SSM and HVPG for HVPG less than 10 mmHg and no correlation between LSM and HVPG. In this study, a cut-off of 3.51 m/s was set to rule out clinically important hypertension and HRVs (sensitivity 97.1% and 93.8%); no HRVs were seen for the cut-off 3.36 m/s and no EVs were observed when the SSM cut-off was of 3.10 m/s.

SS, as determined via pSWE, has also been shown to be predictive of the development of OV; in a study of 78 patients with chronic liver disease conducted by Attia et al., it was used to identify an HVPG of ≥10 mmHg and ≥12 mmHg with high diagnostic performance [31].

#### 2.2.3. Magnetic Resonance Elastography (MRE)

Talwalkar et al. reported that studies in healthy volunteers and patients with various aetiologies of chronic liver disease indicate that SS, as measured via MRE, correlates with PLT count, spleen volume, and splenomegaly, and that an SS of ≥10.5 kPa is associated with OV [32]. A prospective study by Kennedy et al. evaluated the diagnostic performance of MRE of the spleen and liver compared to shear wave elastography in predicting the risk of developing CSPH, using HVPG as a reference. The study prospectively enrolled 36 patients with mixed aetiology (not only viral patients) and SSM via MRE showed the highest correlation with CSPH and the best performance for the diagnosis of CSPH [33].

### 2.3. Serum Markers

There are other non-invasive tools for PH: serum levels of extracellular matrix (ECM)-degraded products [34]; the PLT count, which could be use with spleen diameter for better accuracy [35]; Von Willebrand factor antigen (vWF-Ag), whose value > 315% is associated with higher mortality in patients with compensated and decompensated liver disease [36,37]; the vWF-Ag/thrombocyte ratio (VITRO), which is correlated with transplant-free mortality and decompensation [38]; FibroTest, which is associated with HVPG and with the level of PH (this correlation is greater in patients who have liver disease than in patients with liver cirrhosis) [39]; APRI, which showed a moderate correlation with HVPG [40]; the Cirrhosis probability in Hepatitis C (Lok index—a composite score including PLT, AST, ALT, and INR), which has been independently correlated with the degree of PH and is predictive of CSPH and OV [41]; FIB-4, which has been used for detecting cirrhosis in patients with cACLD [42]; the enhanced liver fibrosis (ELF) score, which is associated with HVPG up to values ≤ 20 mmHg (an ELF score ≥ 11.1 finds patients at high risk of CSPH) [43]; and the indocyanine green 15-min retention (ICG-r15) test, which is a quantitative assessment of liver function that can detect CSPH, rule out the presence of OV, and predict events of decompensation in patients with cACLD [44,45].

Among these NITs, the vWF-Ag/thrombocyte ratio (VITRO) is one of the more promising tools. Jachs et al. conducted a study assessing the role of VITRO for the detection of CSPH (HVPG > 10 mmHg) in patients with available LSM and HVPG data [46]. This study enrolled 302 patients, with cACLD with viral aetiology (50.7%) being the most common. VITRO showed the highest diagnostic accuracy for CSPH, both in the derivation and the validation cohort. VITRO ≤ 1.5 and ≥2.5 ruled out (sensitivity, 97.7%; negative predictive value, 97.5%) and ruled in (specificity, 94.7%; positive predictive value, 91.2%), respectively, CSPH in patients who were ‘unclassifiable’ by Baveno VII criteria, demonstrating that the sequential application of the Baveno VII criteria and VITRO cut-offs decreases the number of previously ‘unclassified’ patients by almost 75% while maintaining PPV and NPV values of >90%, despite the marked reduction of ‘unclassified’ patients. In addition, in patients allocated to the CSPH “ruled out” category, the risk of decompensation at 5 years is negligible, which allows these patients not to be started on preventive treatment [46].

VITRO was also evaluated in a retrospective study also including patients with Child–Pugh B-class cirrhosis from different aetiologies [47]. The VITRO score was significantly higher in patients with CSPH (3.21 versus 1.29 in patients without CSPH) and in patients with OV and ascites (both *p* < 0.0001). Moreover, there was a significant difference in the mean VITRO scores within the Child–Pugh score classes. The VITRO score showed an AUROC of 0.86 (95% CI 0.81–0.91), a sensitivity of 80%, and a specificity of 70% at a cut-off > 1.58 with a positive predictive value (PPV) of 93.2 and a negative predictive value (NPV) of 40.1 in predicting CSPH, resulting in the second-best tool for detecting CSPH, after LSM via TE. The combination of LSM via TE and the VITRO score was able to improve the accuracy of VITRO score alone (*p* = 0.001). These results suggest that VITRO score could be used to detect CSPH and to predict its complications including decompensation events when HVPG and TE are not available.

## 3. NITs for Predicting PH in Patients with HBV-Related cACLD

Despite awareness campaigns, vaccinations, and efficient therapies, HBV infection is still a global health problem, with about 240 million people who are chronic HBV surface antigen (HbsAg) carriers and with the incidence of liver cirrhosis ranging from 8% to 20% in patients who have not been treated. The natural history of HBV infection foresees four phases (HBeAg positive chronic HBV infection, HBeAg positive chronic hepatitis B, HBeAg negative chronic HBV infection, and HBeAg negative chronic hepatitis B) [48]. In patients with HBV-related cirrhosis, the main therapeutic strategy contemplates the use of high barrier resistance nucleos(t)ide analogue (NA) therapy, including entecavir (ETV), tenofovir disoproxil the fumarate, and tenofovir alafenamide. The primary endpoint of therapy is getting viral suppression because it leads to the reduction of chronic necroinflammation, progressive fibrotic liver processes, and risk of hepatocellular carcinoma (HCC) [49].

Table 2 summarizes the main characteristics and findings of the studies evaluating NITs for the prediction of PH in patients with HBV-related cACLD that have been reviewed and that will be discussed in the following sections.

### 3.1. Changes of LSM by TE during Antiviral Treatment

There are several studies that assessed the changes in LSM after starting NA therapy. Lazar et al. conducted a retrospective single-center study including 87 patients with HBV-related cirrhosis who underwent LSM before the start of NA therapy and after 64 months of follow-up, showing a significant decrease in the mean LSM values from the measurement before the onset of therapy versus the second round of therapy (11.7 ± 8.4 kPa vs 8.5 ± 4.1 kPa, *p* = 0.001), while a post hoc analysis showed the decreases after the second year of treatment was not significant [52].

A multicenter, retrospective real-word Canadian study reflects the improvement of LSM values after therapy with NA [53]. The study population of 465 patients was treatment naive and 22% of it was made by cirrhotic patients; the follow-up was up to 5 years and the patients were treated with lamivudine (LAM) or tenofovir (TDF). In patients treated with TDF, there was a LSM regression of −4.2 kPa; in those treated with LAM, the regression was of −1.6 kPa from baseline (*p* < 0.05). Even if the data are retrospective and the documentation is not complete, being a real-word study, it shows that viral suppression, even without HbsAg loss, leads to improvements in inflammation and liver fibrosis; fibrosis could be monitored using NITs like LSM [53]. Moreover, Kim et al. performed a prospective study in South Korea where 121 patients (52% of them were cirrhotic patients) took ETV and were monitored for 3 years using LSM and biochemical exams at baseline and during the follow-up [54]. The median baseline of LSM was 14.3 kPa and the authors showed a mean decrease to 7.3 kPa after 3-year ETV therapy (*p* < 0.001). LSM was able to predict the progression of PH and the onset of decompensation: the reduction in fibrosis and necroinflammation, demonstrated by the decrease is LSM, were associated with reductions in the forthcoming development of liver-related events [54]. Indeed, in a retrospective study on 337 patients with HBV, Xu et al. compared LSM to other NITs (APRI, FIB-4, and GPR) for detecting the liver fibrosis stage, using repeated liver biopsies as the gold standard [56]. They showed that a decrease in LSM values is correlated to a regression of liver fibrosis on histology. The AUROC of the decreased rate of LSM values was higher than that of APRI, FIB-4, and GPR for the prediction of liver fibrosis (0.78, 0.56, 0.55, and 0.57, respectively), demonstrating the usefulness of LSM for non-invasive monitoring of liver fibrosis [56].

### 3.2. Prediction of High-Risk Varices (HRV)

#### 3.2.1. Serum Biomarkers

Different prediction rules have been explored for the prediction of HRV in patients with hepatitis B.

Yan et al. developed and validated a risk scoring system for screening HRV in patients with HBV-related compensated advanced chronic liver disease (cACLD) based on routine laboratory tests and routine liver Doppler ultrasonography [57]. Their algorithm included albumin, PLT count, and portal vein diameter (PVD) and the scoring system was named the albumin–platelet–portal vein diameter varices risk score (APP score). In the training cohort, 221 patients with HBV-related cACLD were included. The AUROC of the APP score was 0.90 (95% CI 0.86–0.94) for identifying HRV and the APP score avoided 51.3–56.6% of OGD for screening HRV with a missing rate lower than 5% and negative predictive value higher than 95%. However, it should be considered that it was a retrospective single-center study, and the ultrasound and EGD examination were performed by different operators during daily clinical work. Most importantly, the APP score has not been compared with other NITs, such as Baveno VI criteria or criteria including SSM.

Interestingly, Zhou et al. assessed and compared the accuracy of LSM via TE, PLT count, APRI, FIB-4, and the Lok index for ruling out HRV in the subgroup of patients with HBV-related compensated cirrhosis who did not meet the Baveno VI criteria [41]. A total of 132 patients were included in the study and stratified according to ALT and bilirubin levels. The prevalence of HRV was 20.5% and LSM and ALT emerged as independent predictors of HRV. The study population was stratified in a subgroup of patients with normal ALT and bilirubin (n = 41) and a subgroup with deranged ALT or bilirubin (n = 91). In the first group, 14 patients (34.1%) had HRV and LSM showed an AUROC of 0.821 (95%CI: 0.670–0.923). At a cut-off value of 20.6 kPa, LSM further spared 16/41 (39.0%) of gastroscopies without missing HRVs. PLT, the Lok index, Model of End-stage Liver Disease (MELD) score, APRI, and FIB-4 had no predictive value for HRV. In the second group, the prevalence of HRV was 14.3%, which was significantly lower than in the first subgroup. The AUROC of LSM was significantly lower (0.672) than that of the Lok index (0.814), PLT (0.741), and MELD score (0.735). A Lok index cut-off ≤ 0.5596 could spare 36/91 (39.6%) of gastroscopies without missing HRVs. A PLT > 100 × 10^9^/L could spare 40/91 (43.9%) of gastroscopies without missing HRVs. These results suggest that, in the subgroup of patients with impaired ALT or bilirubin, the Lok index and PLT could be used for identifying patients without HRV and that concomitant liver inflammation could affect the predictive accuracy of LSM in this setting.

#### 3.2.2. Transient Elastography

Wang et al. analyzed, prospectively, 451 patients with HBV-related cirrhosis who were viral-suppressed or who were undergoing viral suppression, by assessing PH via LSM, SSM, and PLT count [50]. Among patients who achieved viral suppression, in patients with favorable Baveno VI status, no HRVs were missed, 59.5% of patients had no OV, and 40% had grade 1 OV, while in patients with unfavorable Baveno VI status, 41.9% of patients had grade 1 OV and 32.6% had HRV, resulting in a lower risk of variceal bleeding event in the group with viral suppression and favorable Baveno VI status (*p* < 0.001; sensitivity 100%; specificity 46.9%; NPV 100%). Moreover, they validated SSM ≤ 46 kPa as a cut-off in ruling out HRV, since 98.3% of patients with SSM under cut-off had low risk varices (LRVs) (sensitivity 95.71%; specificity 65.31%; NPV 98.33%). This sequential model (Baveno VI + SSM) had a sensitivity of 95.71%, specificity of 76.38%, and NPV of 98.57%, missing only 4.3% of HRV in patients with HBV-related cirrhosis who achieved viral suppression (these data were confirmed in the sub-analysis with stratification based on the duration of maintained viral suppression).

A prospective cross-sectional study held in China validated the Baveno VII algorithm (i.e., patients with an LSM ≤ 15 kPa with PLT > 150 × 10^9^/L can avoid OGD) for ruling out HRV in patients with HBV-related cirrhosis [51]. A total of 504 patients were enrolled and underwent LSM, SSM, spleen diameter measurements, and biochemical examination. The authors demonstrated that the Baveno VII SSM@50 Hz algorithm spared a higher number of not-necessary OGDs than Baveno VI (56.7% vs. 39.1%, *p* < 0.001), resulting in a missed HRV rate of 3.8% for Baveno VII and 2.5% for Baveno VI in patients with HBV-related-cirrhosis. Moreover, they showed, using a Baveno VII algorithm with a cut-off value for SSM ≤ 40 kPa, that the Baveno VII SSM@100 Hz algorithm spared more OGDs than the one with SSM@50 Hz, with a sensitivity of 97% and a negative predictive value of 99.4%.

Wang et al. conducted a prospective single-center study with a derivation cohort (n = 236) and a validation cohort (n = 323) of patients with HBV-related cirrhosis who had achieved viral suppression with available baseline LSM and SSM data via ARFI and OGD [58]. The authors validated the performance of LSM < 1.46 m/s and PLT > 150,000/mmc in ruling out HRVs, which allowed them to correctly spare 9.3% of OGDs in the derivation cohort and 14.2% in the validation cohort, in the absence of misclassification of patients without HRVs. Then, they added SSM with a cut-off ≤ 2.28 m/s: in the derivation cohort, two HRVs were misclassified into LRVs, while the remaining forty-four HRVs were observed in patients with SSM > 2.28 m/s, resulting in a HRV misclassification rate of 4.3%. Finally, they combined LSM < 1.46 m/s + PLT > 150,000/mmc + SSM < 2.28 m/s, showing that this model could spare 38.6% and 33.4% of OGDs, with 4.3% and 3.4% of HRVs missed in the derivation and validation cohort.

An observational study based on the database of a multicenter, observational trial, mainly including patients with HBV-related cirrhosis, validated the ARP strategy, based on LSM and SSM via ARFI and PLT count: 576 patients, between 2017 and 2019, were included in the training cohort and 165 patients, between 2015 and 1026, were included in the validation cohort [59]. In the training cohort, the ARP strategy avoided 40.6% of OGDs, while in the validation cohort it avoided 49.7% of OGDs; the misclassification rate was <5%. Moreover, in the validation cohort, the ARP strategy spared 49.7% of OGDs compared to 34.5% of the Baveno VI criteria and there was a reduction in the missed HRV rate too (1.2% vs. 3.5%, respectively).

### 3.3. LSM for Prediction of Liver-Related Events (LREs)

#### Transient Elastography

Patients with HBV-related liver cirrhosis present a 20% cumulative risk of decompensation in 5 years [48]. A prospective study on 162 patients with HBV-related liver cirrhosis evaluated LSM as a tool to predict LREs. LSM was valuated at baseline, during the course of ETV therapy and at the end of study [60]. During the 2-year ETV treatment, fifteen patients developed LREs (three cases of decompensation, ten of HCC, and two of decompensation and HCC). Multivariate analysis demonstrated age and LSM as independent predictors of LREs. The authors used a time-dependent ROC curve analysis to establish the optimal cut-off of LSM: the baseline cut-off value was calculated as 12.0 kPa with an AUROC of 0.736 (95% CI, 0.620–0.852; *p* = 0.003; sensitivity 93.3%; specificity 42.2%). The population was stratified based on this cut-off and the cumulative incidence rates of LREs was higher in patients with LSM value > 12.0 kPa than those with LSM ≤ 12 kPa (log-rank test, *p* = 0.008). Moreover, to evaluate risk assessment of LRE development according to changes in LSM values, the authors stratified the 157 patients with LRE after 1-year ETV treatment into four groups: (1) baseline LSM of 12 kPa and 1-year LSM 10.3 kPa; (2) baseline LSM ≤ 12 kPa and 1-year LSM ≤ 10.3 kPa; (3) baseline LSM ≤ 12 kPa and 1-year LSM ≥ 10.3 kPa; (4) baseline LSM ≥ 12 kPa and 1-year LSM ≥ 10.3 kPa. The overall incidence of LREs was significantly different among the four groups (*p* = 0.03). This study suggested that serial LSM can be used as a dynamic indicator of the risk of LRE development.

Wu et al. conducted a post hoc analysis of two multicenter, open-label, randomized controlled trials on treatment-naive patients with HBV-related compensated liver cirrhosis, including 438 patients treated with ETV 0.5 mg/die for two years [61]. The primary outcomes were LREs (events that led to hepatic decompensation), development of HCC, and liver-related death. At the end of the follow-up period (104 weeks), thirty-three patients developed LREs: sixteen decompensated cirrhosis complications, eighteen HCC, and three liver-related deaths. Univariate analysis showed a significant association between LREs and the percent change in LSM at 26 weeks from baseline. In patients without LREs, the median LSM value was 17.8 kPa, 10.6 kPa, and 10.2 kPa at week 0, 26, 52, and 78 with two phases of LSM decreasing: the first one was a rapid decrease within the first 26 weeks after treatment with 30.9% down from baseline LSM; the second one was a slowly decreased LSM from week 26 to week 104 after treatment. On the other hand, among 33 patients with LREs the median LSM value was 20.9 kPa, 18.6 kPa, and 20.3 kPa at week 0, 26, 52, and 78; in these patients, there was only a decrease in LSM of 11% in the first 26 weeks and an increasing trend of slowly decreasing after 26 weeks. Moreover, for LSM over 26 weeks, every 10% increase from baseline adjusted the risk of LREs by an increase of 9.3%, which suggests a dynamic change in LSM during the first 26 weeks is an important predictor of LRE during antiviral therapy.

## 4. NITs for Predicting PH in Patients with HCV-Related cACLD

Hepatitis C virus infection is one of the leading causes of chronic liver disease worldwide, with 56.8 million infections [62].

Today, DAAs offer the possibility of treatment to almost the entire infected population, irrespective of the stage of cirrhosis and associated serious comorbidities, always maintaining a high efficacy and tolerability. The efficacy of these drugs is close to 97% in only 3 months of treatment. SVR leads to improvements in liver necroinflammation and regresses hepatic fibrosis. The risk of hepatic failure, HCC, and liver-related mortality is significantly reduced but not eliminated in patients with cirrhosis who clear HCV [63].

The main goal of anti-HCV therapy in patients with compensated cirrhosis is the prevention of decompensation, while in decompensated cirrhosis (Child–Pugh B or C) the aim is to achieve improvement in liver function, possibly leading to recompensation [64]. Several studies have shown significant improvements in bilirubin, albumin, and INR values and, consequently, in MELD and Child–Pugh scores in about one-third to one-half of patients with decompensated cirrhosis in Child–Pugh class B and C after SVR [65,66,67].

Table 3 summarizes the main characteristics and findings of the studies evaluating NITs for the prediction of PH in patients with HCV-related cACLD that have been reviewed and that will be discussed in the following sections.

### 4.1. Prediction of CSPH

#### 4.1.1. Transient Elastography

HVPG measurement remains the gold standard for the diagnosis and the staging of PH, but LSM via TE is recommended by recent Baveno consensuses as an NIT for the identification of patients with cACLD and CPSH and for CSPH risk stratification [14,15].

An Italian multicenter study included sixty-one consecutive patients with HCV cirrhosis with paired HVPG measurement and LSM [71]. The prevalence of CSPH was 77.05% and HVPG > 12 mm Hg (defined as severe PH) was present in 57.38% of patients. In patients with HVPG < 10 mm Hg or <12 mm Hg, a significant correlation between HVPG and LSM (r = 0.81, *p* = 0.0003 and r = 0.91, *p* < 0.0001, respectively) was observed. Conversely, the correlation with LSM hardly reached statistical significance in patients with severe PH. LSM > 13.6 kPa was associated with NPV of 92% with a sensitivity of 97% for the prediction of HVPG > 10 mm Hg; only one out of thirteen patients with LSM below 13.6 kPa had CSPH but were without OV at endoscopy. LSM > 17.6 kPa showed a NPV of 91% with a sensitivity of 94% for the prediction of patients with HVPG > 12 mm Hg and only two (9.09%) out of twenty-two patients with LSM lower than 17.6 kPa displayed severe PH; and only one of them was affected by small OV. Importantly, large OV was not observed among the few false-negative cases. This study has demonstrated that the correlation between LSM and HVPG values higher than 10 mmHg is excellent in patients with HCV-related cirrhosis.

#### 4.1.2. Changes of LSM by TE after SVR

The achievement of SVR via DAAs represents a crucial event in the history of patients with HCV infection and marks a significant change status. Although studies evaluating the fibrosis stage in liver biopsies after SVR in HCV-advanced chronic liver disease (ACLD) patients are lacking and histological changes are not well known, a retrospective study conducted on 214 Japanese patients with HCV genotype 1b who received 24-week daclatasvir and asunaprevir dual therapy showed that LSM significantly decreased at 24, 48, and 72 weeks after the end of treatment, compared to the baseline [72]. However, the improvement in necroinflammation after SVR can rapidly affect LSM, leading to a decrease in its accuracy and correlation with the fibrosis stage [73]. Given that the best TE cut-off values are significantly lower after SVR and they are less reliable, the use of LSM by TE for discriminating different liver fibrosis stages after SVR is controversial [74].

Dynamic changes of LSM over time can be useful to predict CSPH, rather than changes in fibrosis stage. Lens et al. performed a prospective multicenter study that included 226 patients with HCV-related cirrhosis and CSPH, defined as HVPG > 10 mmHg [55]. The study evaluated changes in HVPG and LSM after 96 weeks from SVR 96. At week 96 after eradication, 47% of patients no longer had CSPH. A total of 65% continued to have CSPH. However, the percentage of patients with HVPG > 16 mmHg was reduced from 41% to 15%. The LSM of these patients at baseline was 26.2 kPa and significantly reduced after 24 weeks from SVR (−8.7 ± 1.2 kPa). After 96 weeks from SVR, it was reduced compared to SVR 24 by −2.3 kPa. The study failed, however, to demonstrate a statistically significant correlation between the use of DAAs and the reduction in both HVPG and LSM, suggesting that the hepatic hemodynamic is influenced by several factors.

### 4.2. Prediction of HRV

#### 4.2.1. Serum Markers and Transient Elastography

As discussed above, Baveno VI criteria are commonly used for ruling out HRV, given that they allow researchers to identify patients with a low risk of having HRV with an acceptable rate of missing HRV. However, alternative NITs, not including LSM via TE, have been evaluated.

Particularly, the REAL study included 381 patients with HCV-related cirrhosis [68]. Using multivariate logistic analysis, laboratory variables were selected to determine which were independently associated with medium/large EVs and RESIST-HCV criteria have been developed in order to identify a subgroup of patients with a low risk of HRV. Medium/large OVs were identified in five of two-hundred sixteen patients (2.3%) using the Baveno VI criteria and in thirteen of four-hundred ninety-seven patients (2.6%) using the Expanded Baveno VI criteria. The PLT count and albumin level were independently associated with medium/large OVs: the best cut-off values were 120 × 10^9^ cells/μL for the PLT count and 3.6 g/dL for the serum albumin level. Therefore, patients were classified as RESIST-HCV low risk (low probability of HRV) if the PLT was >120,000 mmc and serum albumin was >3.6 g/dL or RESIST-HCV high risk if the PLT was <120,000 mmc and/or serum albumin was <3.6 g/dL. In the training cohort of 326 patients, 119 (36.5%) met the RESIST-HCV criteria and the NPV was 99.2%. Among 1055 patients in the validation cohort, 315 (30%) met the RESIST-HCV criteria and the NPV was 98.1%. Adding TE to the RESIST-HCV criteria reduced unnecessary OGDs for approximately 25% of patients and the NPV was 98.2%.

The similar performance of biochemical-based criteria (i.e., RESIST-HCV criteria) compared to LSM-based criteria (i.e., Baveno VI criteria) has been also confirmed in a multi-aetiological cohort (including about 33% of patients with HCV) of 1657 patients. This study compared different NITs by using decision curve analysis and it showed that, at a threshold probability of 5% of missing HRVs, the Baveno VI criteria showed the maximum net benefit; the RESIST-HCV criteria were the next best, with the need for ninety-five additional elastographies to detect one additional varix needing treatment (VNT) [8]. In light of similar performance, it should be considered that the RESIST-HCV criteria do not need LSM via TE for risk stratification and they do not need patient permission to access to the liver unit to perform LSM, therefore representing the ideal alternative to LSM-based criteria, especially in resource-limited settings.

Both the Baveno VI and RESIST-HCV criteria have been developed and validated in patients with active HCV viremia. With the worldwide diffusion of DAA therapy, tools to predict the development of HRV after SVR are needed.

In this regard, Baveno VII consensus recommends that, after HCV eradication, patients with a normal liver ultrasonography and invasive or non-invasive tests excluding advanced fibrosis do not need follow-up, while patients with advanced fibrosis (F3) and especially patients with compensated cirrhosis (F4) should undergo ultrasound surveillance for HCC, because the risk of HCC is still present despite HCV being cured [15]. However, patients who achieved a consistent decrease in LSM < 12 kPa with a PLT count > 150,000/mmc (i.e., Baveno VII criteria) have a negligible risk of decompensation after HCV eradication, in absence of other risk factors for liver disease (alcohol abuse, obesity and/or type 2 diabetes, etc.) [15].

RESIST-HCV criteria have also been evaluated in this setting, with the aim of predicting progression to HRV after HCV eradication via DAAs in patients with cACLD, who had no OV or only a small OV before treatment. A prospective multicenter study enrolled 353 patients in Child–Pugh class A at three referral centers with a mean follow-up of 44.2 months. The RESIST-HCV criteria showed the highest AUROC (0.70, 95% CI 0.65–0.75), correctly sparing the highest number of OGDs (54.3%), with the lowest false-positive rate (45.7%), compared with the elastography-based criteria (Baveno VII criteria). More importantly, the net benefit evaluated using decision curve analysis was higher for the RESIST-HCV criteria, compared to the stiffness-based criteria [9].

A prospective study conducted in 2020 at two Italian centers assessed the changes in OV status in 63 patients with compensated advanced chronic liver disease (cACLD) who achieved SVR after DAA treatment [69]. The diagnostic performance of non-invasive predictors of OV such as Baveno VI, Expanded Baveno VI criteria, and PLT count/spleen diameter ratio (PLT/SDR) and the number of endoscopies spared via their application were evaluated. At baseline, the application of the Baveno VI and expanded BVI criteria would have saved seven (11.1%) and seventeen (27.0%) endoscopies, missing OVs in one patient each (14.3% and 5.9%, respectively), while application of the PLT/SDR would have saved twenty-six endoscopies (41.3%), missing varices in three patients (11.5%). After follow-up, the application of Baveno VI and expanded Baveno VII criteria would have saved seventeen (30.4%) and twenty-four (42.9%) endoscopies, missing OVs in two (11.9%) and four (16.7%) patients, respectively, and the application the PLT/SDR would have spared thirty-one endoscopies (55.4%), missing OVs in seven patients (19.4%). Notably, at baseline, none of the patients with HRV would have been misclassified using any parameter, while at follow-up HRV would have been missed in one patient (3.2%) using the PLT/SDR and in none using either the Baveno VI or expanded Baveno VI criteria.

#### 4.2.2. Share Wave Elastography

A prospective study by Han et al. assessed 60 patients with cirrhosis and 20 healthy volunteers [75]. The most common aetiology was HCV infection, in 80% of HCV cases. Shear wave elastography revealed elevated liver rigidity among cirrhotic individuals (ranging from 13.1 to 58 kPa). This imaging modality effectively discriminates between cirrhotic patients with gastro-oesophageal varices and those without, utilizing a threshold of 26.5 kPa and achieving an 88% sensitivity and 85% specificity. Furthermore, shear wave elastography indicated a direct correlation between liver stiffness and the variceal size increment (ranging from 47.3 to 49.5 kPa in F3) [75].

### 4.3. Prediction of LREs

#### 4.3.1. Serum Markers

A retrospective study of 321 patients with HCV-related cACLD assessed the performance of NITs for predicting the development of LREs after SVR via DAAs [70]. LREs were defined as the development of decompensation from PH and the occurrence of HCC. After a median follow-up of 48 months, LREs occurred in 13.7% of patients (10% PH-related decompensation and 3.7% HCC); FIB-4 was the only predictor among the NITs evaluated of PH-related decompensation, with cut-off values of 2.03 and 2.21 at 1 and 2 years after SVR, respectively. Older age, genotype 3, diabetes mellitus, and FIB-4 before and after SVR emerged as predictors of HCC occurrence. The results of this study highlight that, although the incidence of LREs decreases after DAA treatment, patients with cACLD remain at risk of developing liver complications even after SVR. FIB-4 calculation before and after SVR can be useful to predict this risk of hepatic decompensation and HCC occurrence. The low risk of hepatic decompensation, especially in patients without HRV at baseline, has been recently confirmed in a prospective Italian study, showing a 4-year cumulative risk of decompensation lower than 1% [76].

#### 4.3.2. Transient Elastography

The performance of NITs, including LSM via TE, for predicting LREs has been prospectively evaluated in a two-center study including 572 patients with cACLD who achieved SVR after DAA treatment [77]. After a median follow-up time of 2.8 years, the incidence rate of PH-related decompensation was 0.34/100 patient years. Interestingly, all patients who developed decompensation had a baseline LSM > 20 kPa, in the absence of a significant improvement in LSM during follow-up in most of them. Albumin levels and LSM < 10 kPa, both evaluated at follow-up, were independently associated with the risk of HCC. The combination of these two predictors allowed the identification of patients with LSM ≥ 20 kPa at follow-up or those with LSM between 10–20 kPa and albumin levels < 4.4 g/dL, who were at the highest risk of HCC.

## 5. Discussion

HBV and HCV infection still represent leading main aetiologies of chronic liver disease and they are potentially associated with the progression to cirrhosis and HCC. Therefore, it is necessary to start antiviral therapy as soon as possible to achieve viral suppression or eradication and to possibly reverse liver dysfunction. Several studies have demonstrated that antiviral therapy can modify the natural history of viral hepatitis, reducing liver fibrosis and, therefore, the risk of developing cirrhosis.

LSM is useful to assess and monitor the stage of liver fibrosis and it is possible to observe a decrease in LSM values during therapy. Therefore, LSM could represent a good surrogate for liver biopsy, as it is not invasive, in patients with untreated viral hepatitis [52,53,60]. Moreover, it must be considered that the spectrum of the severity of liver fibrosis is a continuum and that LSM has a limited ability in evaluating consecutive stages of fibrosis. This is the reason why, from a clinical point of view, it appears to be more relevant to rule-in or rule-out a disease or a prognostic stage, rather than to provide a precise stage of liver fibrosis [14,15].

Identification of HRV and CSPH represents a crucial point in risk stratification of patients with cACLD, because it is associated with a significantly higher risk of hepatic decompensation and death [78]. The presence of OVs is an unequivocal sign of CSPH and the gold standard for detecting them is an OGD, which is an invasive test.

LSM has demonstrated good diagnostic accuracy for ruling in and ruling out cACLD and CSPH in patients with untreated HBV or HCV infection and the so-called “rule of 5” is recommended by the Baveno VII consensus, as well as by recently published American Association for the Study of Liver Disease (AASLD) practice guidance [79]. When combined with the PLT count, LSM is an useful tool for the identification of patients that can avoid OGD. Therefore, when TE is available, patients with untreated HBV or HCV infection should be stratified according to the Baveno VI criteria, which is a validated tool to rule out HRV and avoid endoscopic screening. When TE is not available or not feasible, the use of biochemical-based NITs (for example, the RESIST criteria [68]) is a reasonable alternative in both HCV- and HBV-infected patients [8].

However, it should be noted that, in the setting of HCV patients after SVR via DAAs, the percentage of LSM decrease did not accurately predict fibrosis regression [80] and, therefore, international guidelines recommend against the use of NITs (including LSM via TE and other elastography techniques) to detect fibrosis regression after SVR in HCV patients [16,74]. Although Baveno VII consensus has proposed different thresholds of TE and PLT count to predict HRV after SVR [15], this algorithm has been shown to be not superior to the easy-to-use biochemical-based RESIST criteria, with these latter criteria performing well and being validated also for the prediction of the progression to HRV after SVR [9].

On the other hand, SSM seems to correlate better with CSPH and decompensation of cirrhosis, compared to LSM, and it is not affected by factors like steatosis, liver congestion, or cholestasis. SSM is able to correctly spare OGDs with a residual risk of missing HRVs lower than 5%, which is the threshold conventionally considered as safe for NITs. In patients with untreated viral aetiology, SSM can be used to rule-in and rule-out CSPH with the cut-offs of <21 kPa and >50 kPa, respectively, while the cut-off of <40 kPa is useful to optimize the risk stratification among patients classified as having a low risk of HRV, according to Baveno VI, leading to a further reduction in unnecessary endoscopies [15,50,51]. LSM is a good surrogate for liver fibrosis, so it could be useful as first step, while SSM is a good surrogate for PH and may be useful in follow-up [23,27]. Moreover, both LSM and SSM are useful for predicting the onset of liver-related events. Compared to LSM, there are less factors that influence the accuracy of SSM: the main factors that could be difficult are the operator expertise, obesity, ascites, and the localization of the spleen. Given the limited range of spleen stiffness that can be measured with SSM@50 Hz, which has 75 kPa as upper limit, a new probe (SSM@100 Hz) has been recently introduced to overcome this limitation. Although initial data suggest that SSM@100 Hz could spare a higher number of unnecessary endoscopies compared to SSM@50 Hz [51], validation of the best cut-off with the SSM@100 Hz is still needed [15].

Alternative elastography techniques, such as SWE and MRE, represent promising tools for risk stratification of PH and its complications in this setting. Compared to TE, SWE offers improved accuracy and depth penetration and preliminary data suggest that it could be more accurate than TE, resulting in a lower risk of unreliable measurements [19]. On the other side, MRE offers a high accuracy; Because it is not operator-dependent, it is not affected by ascites, obesity, and interposition of the bowel and it is associated with a lower risk of sampling error, compared to TE. Small studies have showed a promising accuracy of MRE for the detection of CSPH [33]. However, both SWE and MRE are limited by the higher costs and the lower availability in clinical practice outside of research protocols. Moreover, MRE strictly relies on the ability of patients to cooperate during the examination.

## 6. Conclusions

Despite the revolutionary introduction of antiviral therapy, which can hamper the progression to liver cirrhosis when started at the right time, the risk of decompensation in patients with viral cirrhosis still persists. Liver biopsy, OGD, and HVPG are the gold standard tests to identify the stage of fibrosis and the presence of CSPH, which determines the risk of decompensation, but they are invasive tests. To detect and treat CSPH, rapid NITs that can be used not only in point-of-care centers but also in non-specialist hospitals are needed. The tests we analyzed in this review are scores based on routine blood tests and instrumental measures such as LSM and SSM. They could speed up the diagnosis of CSPH because they are easy to use, quick to process, and not uncomfortable for patients. Furthermore, these tests are easily repeatable and showed a good correlation with HVPG changes that lead to the progression to cirrhosis, with response to aetiological therapy and other factors such as aetiology, so they represent useful tools for follow-up. Further studies are needed to demonstrate the sensitivity, specificity, accuracy, and the net benefit of novel biomarkers, but routine blood tests and LSM and SSM via TE have been shown to be excellent surrogates in diagnosing and staging PH and in predicting patients’ outcomes.

## Figures and Tables

**Table 1:** Main characteristics of elastography-based techniques.

	Target	Advantages	Limits
VCTE	- liver - spleen	- medium–low cost; - correlated to progression of liver disease; - dynamic biomarker; - easy and rapid to use; - high sensibility and high specificity.	- dependence on operator experience; - obesity, ascites, and biliary dilation reduce accuracy; - sampling bias.
SWE	- liver - spleen	- higher specificity than VCTE; - higher correlation with HVPG.	- higher costs; - not available in all centers.
MRE	- liver - spleen	- detection of heterogeneous pattern of fibrosis and early stage of fibrosis; - absence of operator dependence.	- respiratory collaboration; - claustrophobia; - high costs.

VCTE, vibration-controlled transient elastography. SWE, shear wave elastography. MRE, magnetic resonance elastography. HVPG, hepatic venous pressure gradient.

**Table 2:** Characteristics and main results of the studies evaluating TE for the prediction of PH.

First Name, Year	Study Design	Number and Clinical Characteristics of Patients	Outcome	NITs Evaluated	Main Performance Measures	Cut-Off
Wang et al., 2021 [50]	Single-center prospective study	451 patients - 351 withvirological suppression; - LSM and SSM via TE and OGD at enrollment.	Ruling out HRV	Baveno VI criteria, SSM	Sensitivity = 95.71%, Specificity = 76.38%, NPV = 98.57% Negative likelihood ratio = 0.06	Baveno VI: LSM < 20 kPa and PLT > 150,000/mmc SSM ≤ 46 kPa
Zhou et al., 2019 [41]	Multicenter retrospective study	132 patients - compensated liver cirrhosis, who did not meet the Baveno VI criteria; - FibroScan procedure and OGD within 6 months; - complete clinical, laboratory, and imaging data.	Prediction of HRV	LSM, PLT, Lok index, MELD, APRI, FIB-4, ALT, bilirubin	41 patients with ALT and TBil < 2 ULN: - LSM AUROC = 0.821. In the 91 patients with ALT or TBiL ≥ 2 ULN: - Lok index AUROC = 0.814; - PLT AUROC = 0.741.	Lok index: 0.4531; LSM: 20.6 kPa; PLT > 151,000/mmc; MELD 6.0.
Zhang et al., 2023 [51]	Prospective, cross-sectional study	504 patients with ongoing antiviral therapy or naïve at enrolment	Prediction of HRV	SSM	Missed HRV rate < 5%	Cut-off = 40 kPa
Lazar et al., 2022 [52]	Single-center, retrospective study	87 patients initiated on NA therapy after being non-responders, or relapses, or naïve to Peg-Interferon or LAM therapy	Stiffness after NA therapy	LSM	*p* = 0.001	From 11.7 ± 8.4 kPa to 8.5 ± 4.1 kPa
Ramji et al., 2022 [53]	Multicenter, retrospective, real-word study	465 patients naïve to therapy - 299 received TDF; - 166 received LAM.	Stiffness after NA therapy	LSM	*p* < 0.05	−4.2 kPa with TDF and −1.6 kPa with LAM
Kim et al., 2014 [54]	Prospective study	121 patients who completed an ETV 3-year treatment	Stiffness after NA therapy	LSM	*p* < 0.001	−7 kPa
Lens et al., 2020 [55]	Multicenter prospective study	226 after DAAs therapy and SVR	Stiffness after DAA therapy	LSM	*p* < 0.05	−11 ± 5.9% at SVR96 additional relative change

NITs, non-invasive tests. LSM, liver stiffness measurement. SSM, spleen stiffness measurement. HRV, high-risk varices. OGD, oesophagogastroduodenoscopy. PLT, platelets. NPV, negative predictive value. NA, nucleos(t)ide analogues. TE, transient elastography. kPa, KiloPascal. LAM, lamivudine. ETV, entecavir. TDF, tenofovir disoproxil Fumarate. tBil, total bilirubin. AUROC, area under the receiver operator characteristics curve.

**Table 3:** Characteristics and main results of the studies evaluating serum biomarkers for the prediction of PH.

First Name, Year	Study Design	Number of Patients	Outcome	NITs Evaluated	Main Performance Measures	Cut-Off
Calvaruso et al., 2019 [68]	Cross-sectional multicenter study	1381: - training cohort of 326 patients; - validation cohort of 1.055 patients.	Prediction of HRV	RESIST-HCV criteria	- Training cohort: NPV = 99.2%; - Validation cohort NPV = 98.2%	RESIST-HCV low risk: PLT > 120,000/mmc and albumin > 3.6 g/dL; RESIST-HCV high risk: PLT < 120,000/mmc or serum albumin < 3.6 g/dL
Sharma et al., 2020 [8]	Single-center retrospective analysis of a prospectively maintained database	1657: - 895 with cACLD; - 762 non-cACLD.	Prediction of HRV	Baveno VI, platelet-albumin criteria	Baveno VI: VNT = 97.3%, NPV = 96.9%	Baveno VI: LSM < 20 kPa and PLT > 150,000/mmc; Platelet-albumin criteria: serum albumin > 4 g/dL and PLT > 114,000/mmc
Giannini et al., 2020 [69]	Multicenter prospective study	63 patients with cACLD and SVR after DAAs therapy	Prediction of HRV	Baveno VI, expanded Baveno VI, platelet count/spleen diameter ratio	Baveno VI NPV = 88.2%; expanded Baveno VI NPV = 83.3%; platelet count/spleen diameter ratio NPV = 80.7%	Baveno VI: LSM < 20 kPa and PLT > 150,000/mmc; Expanded Baveno VI criteria: LSM < 25 kPa and PLT > 110,000/mmc; Platelet count/spleen diameter ratio: 909 (n/mm^3^)/mm
Fernandez-Alvarez P et al., 2023 [70]	Single-center, observational retrospective study	321 patients with cACLD and SVR after DAAs therapy	Prediction of LREs	APRI score, FIB-4 score, LSM	Association between FIB-4 and PH decompensation at 1 year: HR = 1.31, 95%CI (1.15–1.48). Association between FIB-4 and PH decompensation at 2 years: HR 1.42, 95%CI (1.23–1.64)	FIB-4: 2.03 and 2.21
Yan et al., 2021 [57]	Single-center retrospective study	334 patients with cACLD - with persistence of HBsAg for > 6 months and with anti-viral experience; - interval time between the ultrasound and OGD of no more than 6 months.	Prediction of HRV	albumin, PLT, portal vein diameter (APP score)	AUROC of APP score: 0.90 (95% CI 0.86–0.94) for identifying HRV. NPV > 95%.	APP: 0.24

NITs, non-invasive tests. cACLD, compensated advanced chronic liver disease. LSM, liver stiffness measurement. HRV, high-risk varices. OGD, oesophagogastroduodenoscopy. PLT, platelets. NPV, negative predictive value. DAA, direct-acting antiviral agents. SVR, sustained virologic response. TE, transient elastography. kPa, KiloPascal. AUROC, area under the receiver operator characteristics curve. HR, hazard ratio. 95%CI, 95% confidence interval.
